# Retraction: Relationship between acute stress responses and quality of life in Chinese health care workers during the COVID-19 outbreak

**DOI:** 10.3389/fpsyg.2024.1527505

**Published:** 2024-11-25

**Authors:** Frontiers Editorial Office

The journal retracts the 2021 article cited above.

Following publication, concerns were raised regarding the provenance of the patient data used in this study. During an investigation conducted in accordance with Frontiers' policies, the authors confirmed the existence of a conflict over the ownership of the patient data between different research groups. Unauthorized use of data is a violation of Frontiers' guidelines and those of the Committee on Publication Ethics. As such, this article has been retracted.

The retraction was approved by the Chief Editors of Psychology and the Chief Executive Editor of Frontiers. The authors collaborated in the investigation and approved the retraction.

